# Supplementary material for: Dietary stearic acid regulates mitochondria in vivo in humans
Source: Nat Commun. 2018 Aug 7;9:3129. doi: 10.1038/s41467-018-05614-6 (PMC6081440; doi:10.1038/s41467-018-05614-6)
Supplement: Supplementary file 1 — Supplementary Information [file 41467_2018_5614_MOESM1_ESM.pdf]

Supplementary Information

“Dietary stearic acid regulates mitochondria *in vivo* in humans”

Senyilmaz-Tiebe et al.

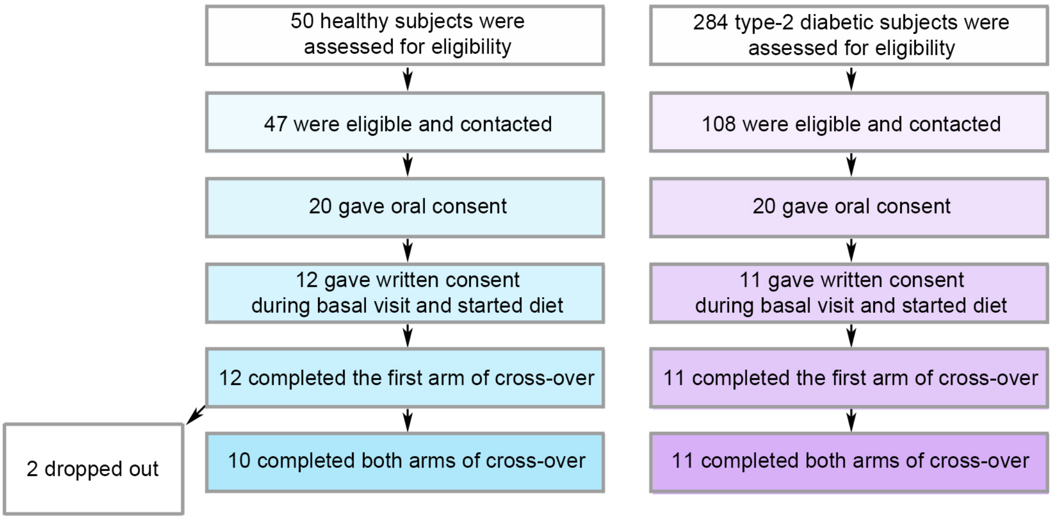

Supplementary Figure 1: Subject baseline characteristics and multivariate regression analysis on mitochondrial morphology.
Overview of clinical study recruitment.

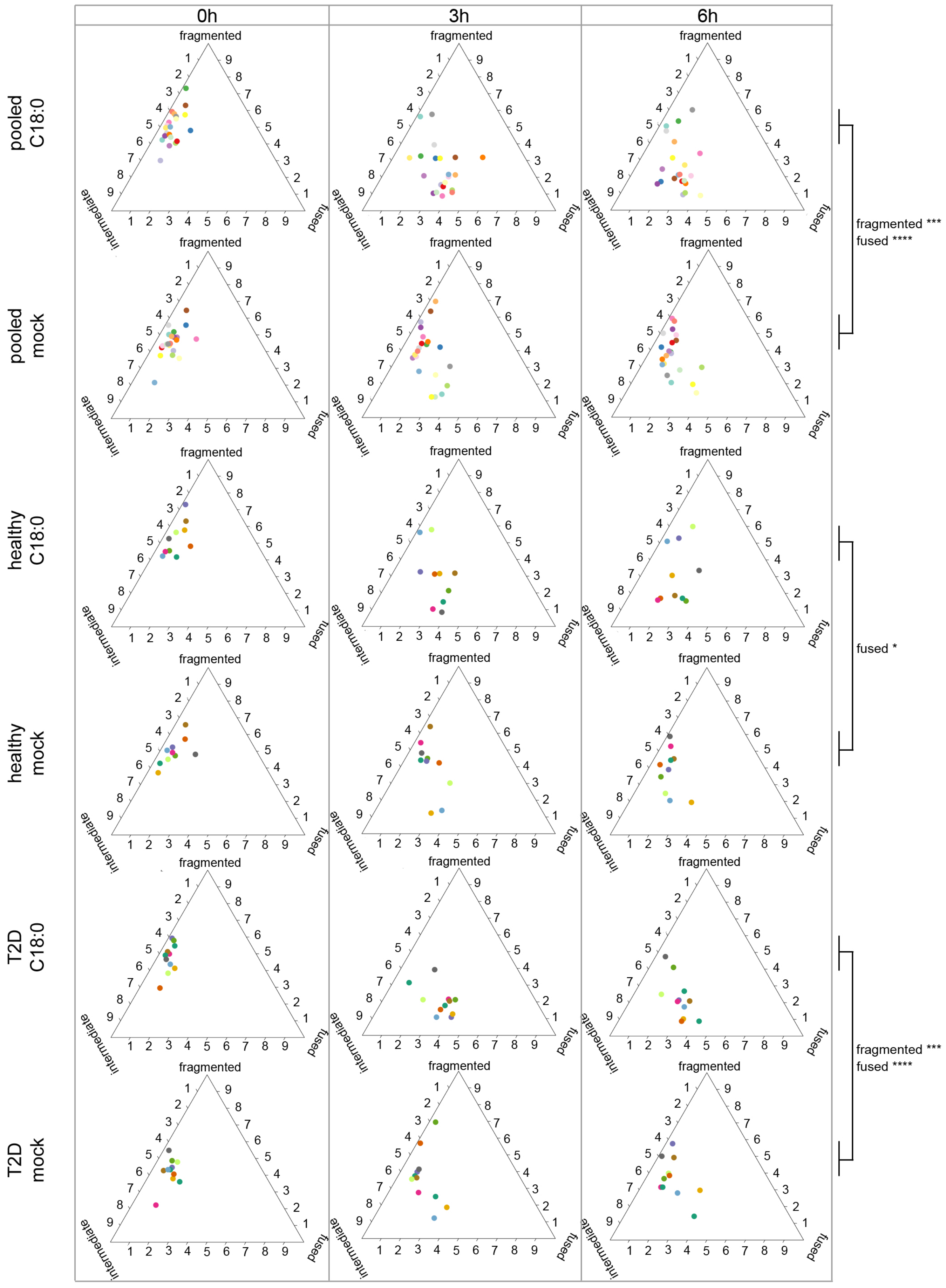

Supplementary Figure 2

Supplementary Figure 2: Statistical analysis using simplex plots on the entire profile of morphology classifications confirms that C18:0 feeding significantly causes mitochondrial fusion in neutrophils compared to the mock drink.
Simplex plots were used to visualize the changes in mitochondrial morphology across timepoints, shown in main Figure 1c-h. Every subject is represented by one dot of one color that remains the same across timepoints. The statistical significance of the difference between the C18:0 and the mock drinks in terms of changes in mitochondrial morphology across timepoints was calculated as described in [25,26]. n=21 subjects, of which 10 healthy and 11 type-2 diabetic. *p<0.05, ***p<0.001, ****p<0.0001. Error bars = std. dev.

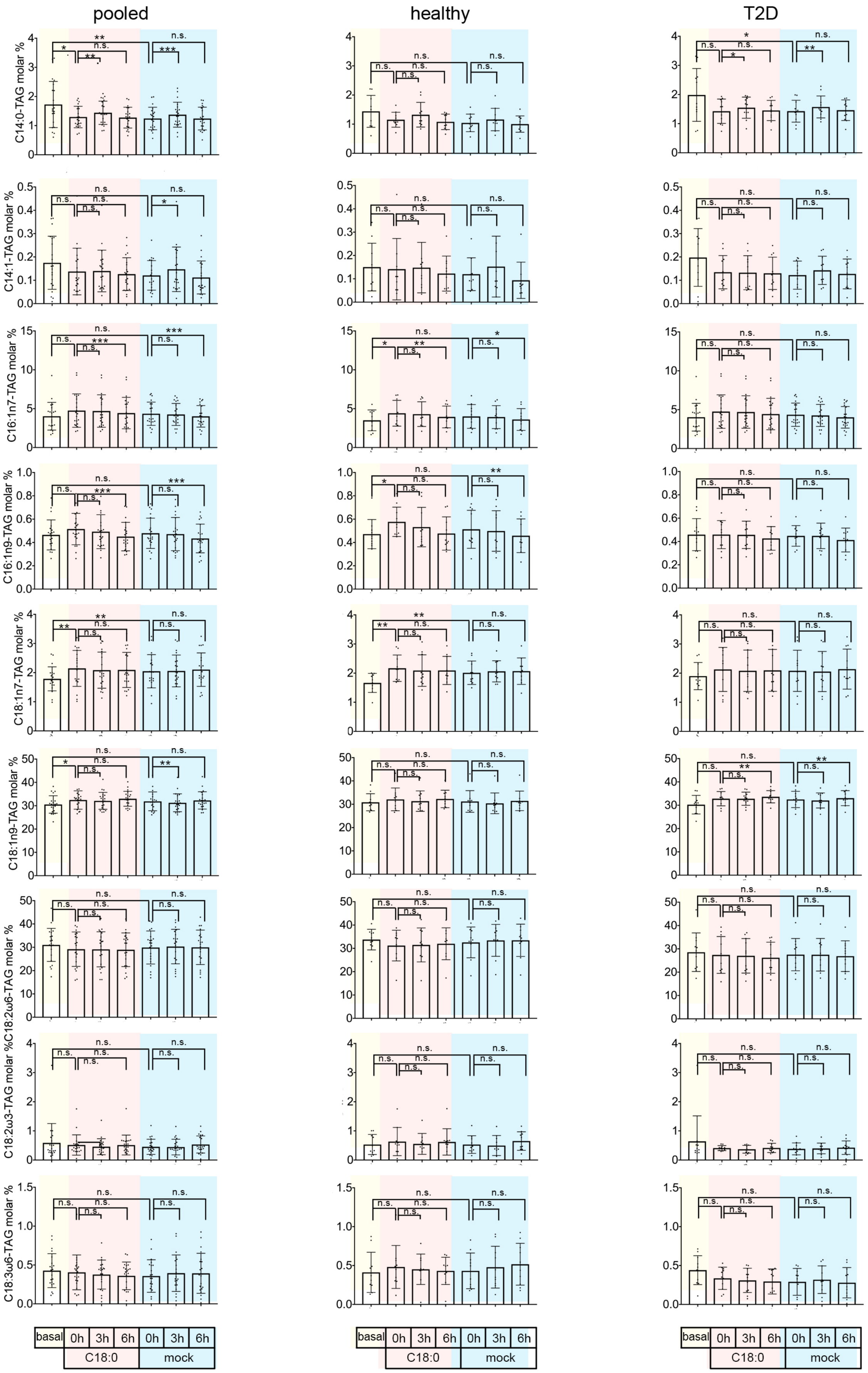

Supplementary Figure 3

Supplementary Figure 3: Serum levels of other fatty acids do not exhibit changes dependent specifically on C18:0 dietary intake.

Levels of multiple fatty acids (C14:0, C14:1, C16:1n7, C16:1n9, C18:1n7, C18:1n9, C18:2omega6, C18:2omega3 and C18:3omega6) from the triglyceride fraction of serum, were measured in parallel to the C18:0-TAG shown in main Figure 2, across all timepoints and nutrient conditions. None of them except C18:0 changes in abundance specifically in response to C18:0 feeding. n=21 subjects, of which 10 healthy and 11 type-2 diabetic. *$p<0.05$, **$p<0.01$ by student t-test. Error bars = std. dev.

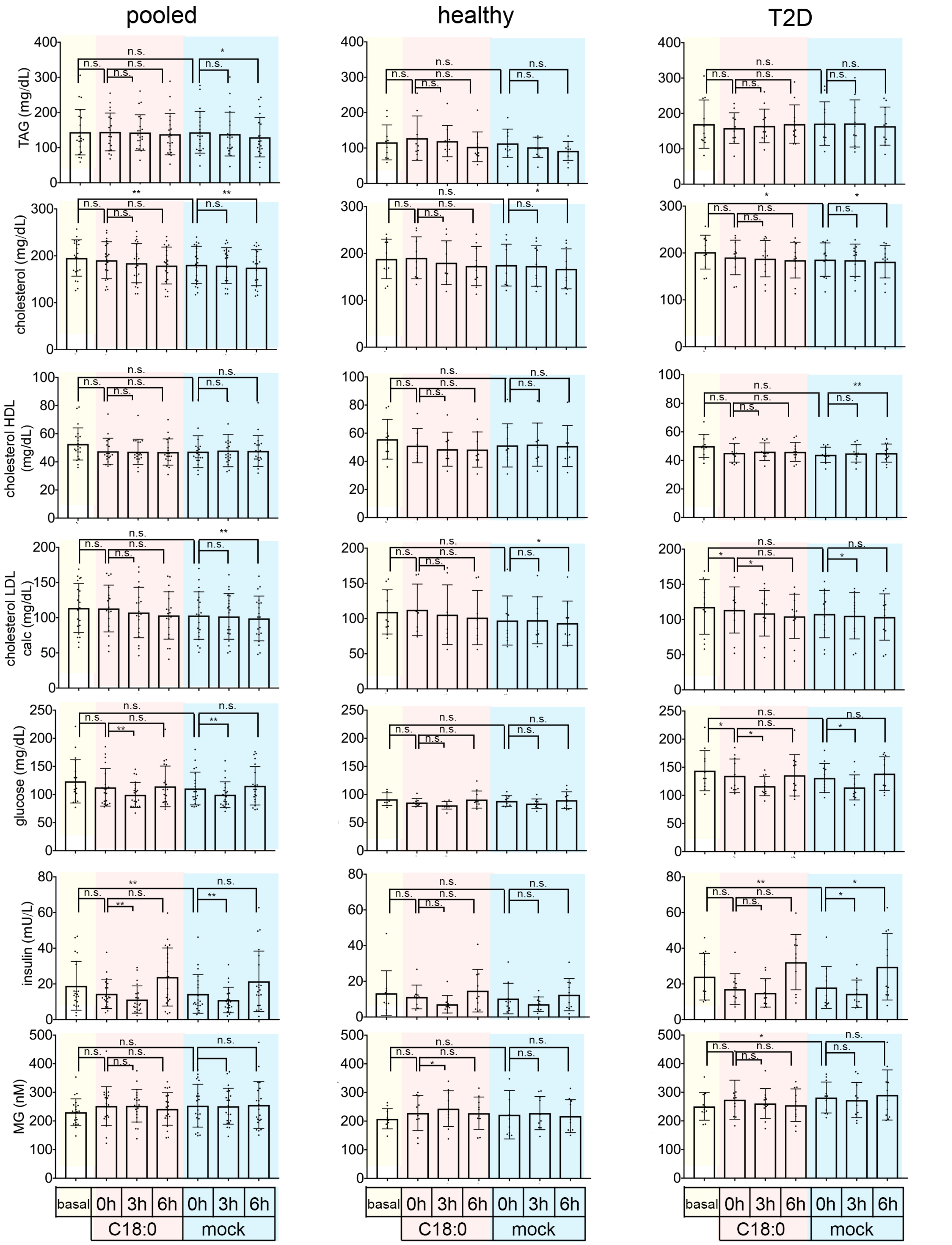

Supplementary Figure 4: C18:0 intake does not affect serum levels of total TAGs, cholesterol, glucose, insulin, or methylglyoxal (MG).
n=21 subjects, of which 10 healthy subjects and 11 type-2 diabetic subjects. Error bars = standard deviation. *p<0.05, **p<0.01 by student t-test.

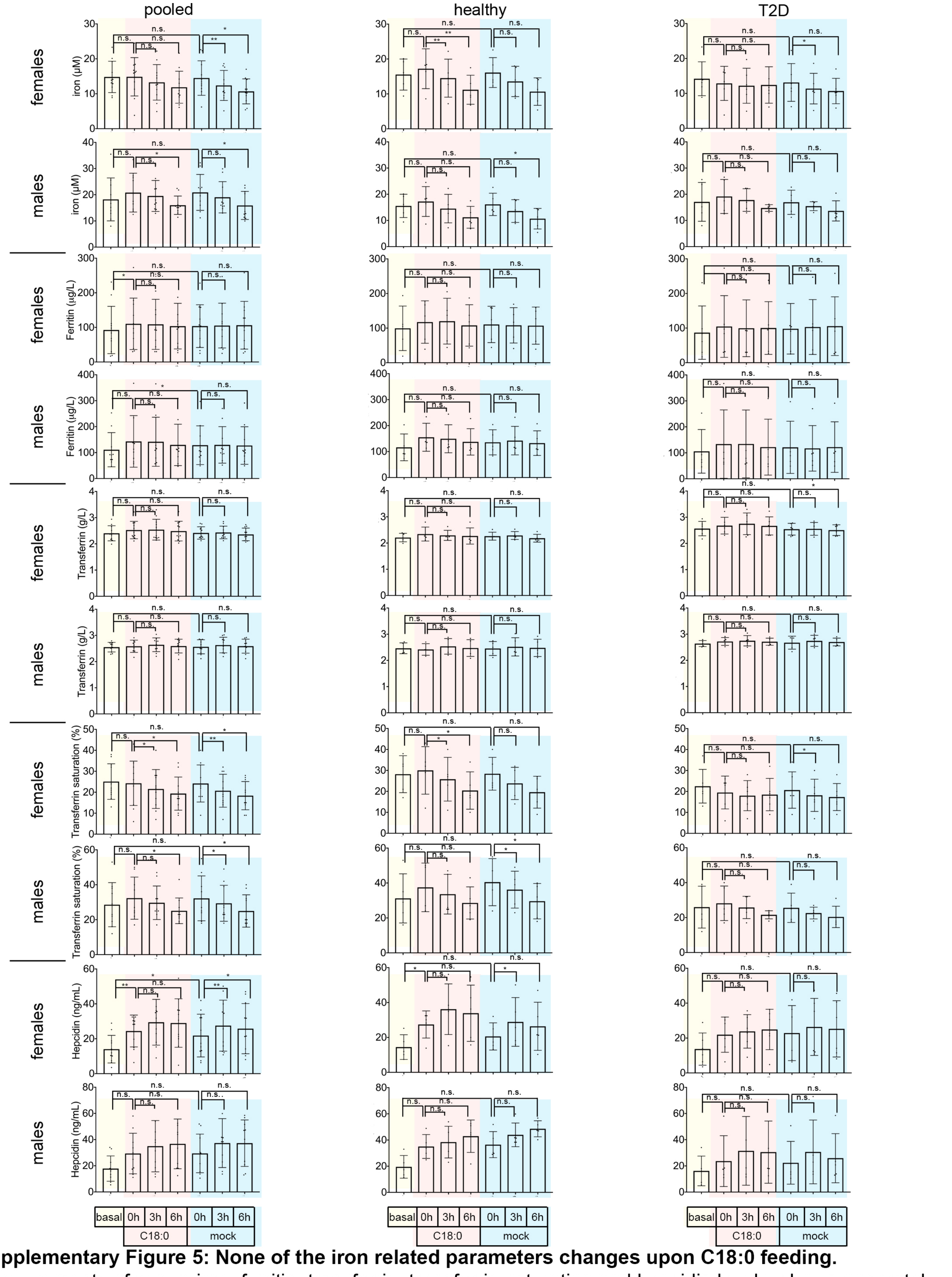

Supplementary Figure 5: None of the iron related parameters changes upon C18:0 feeding. Measurements of serum iron, ferritin, transferrin, transferrin saturation and hepcidin levels, shown separately for males and females due to sexual dimorphism in iron metabolism. n=5 healthy females, 6 type-2 diabetic females, 5 healthy males, and 5 type-2 diabetic males. *p<0.05, **p<0.01 by student t-test. Error bars=std. dev.

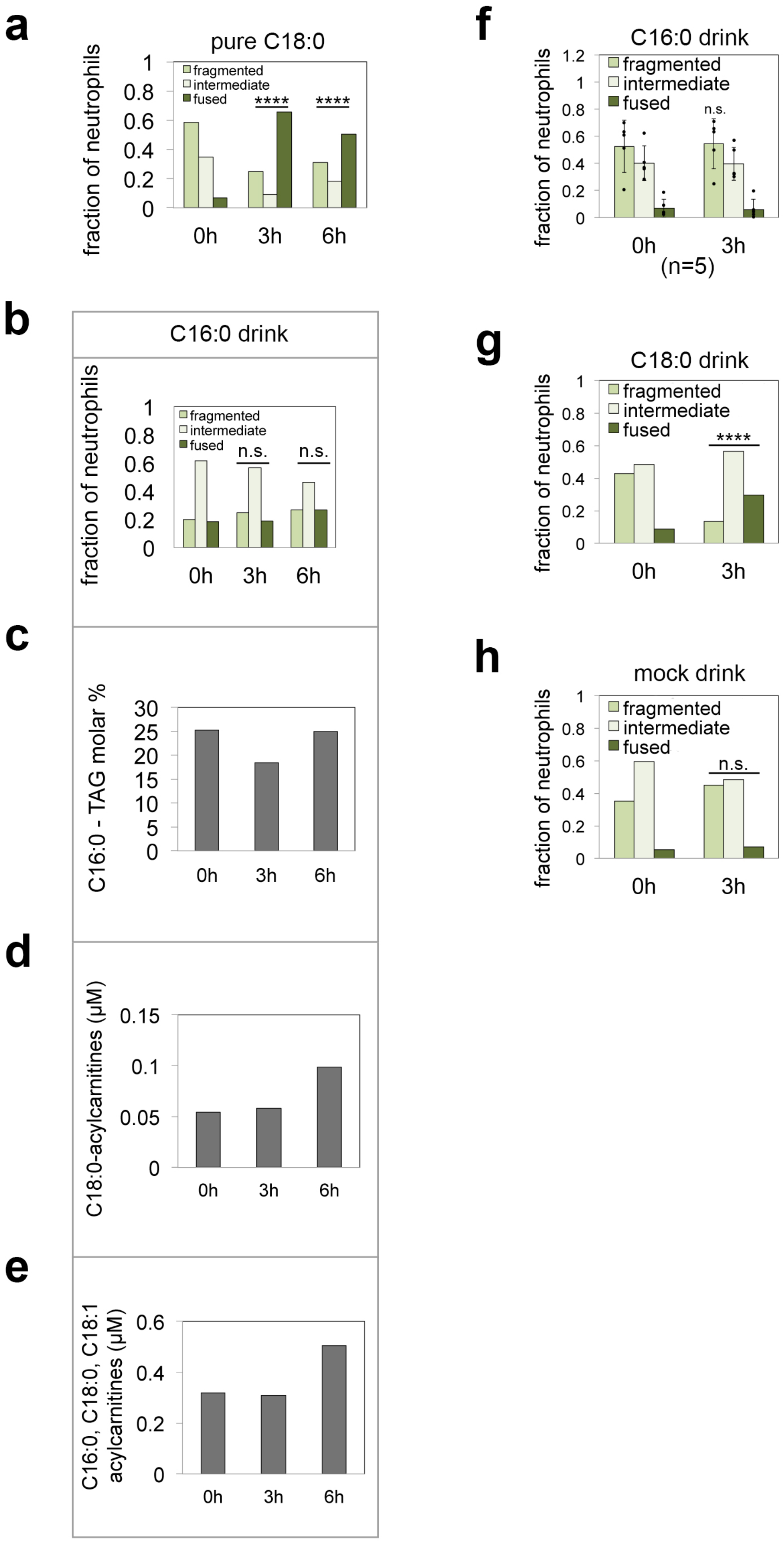

Supplementary Figure 6

Supplementary Figure 6: The effects on mitochondrial morphology of the C18:0 banana milk shake are reproduced by ingesting pure C18:0 (without the banana milk shake) and are not reproduced by ingesting a banana milk shake containing C16:0.

(a) The effect of the C18:0 banana milk shake on mitochondrial morphology is due to the C18:0, because ingestion of pure C18:0 also causes fusion of mitochondria in neutrophils. Twenty-four grams of C18:0, equal to the amount present in the C18:0 banana milk shake, were added to an equal volume of drinking water (250 mL). The mixture was heated up in the microwave to emulsify the fatty acid and the mixture was ingested. Blood was drawn at the indicated timepoints after C18:0 ingestion and the mitochondrial morphology of blood leukocytes was scored as shown in main Figure 1b. (n=60 cells per timepoint, 1 subject. ****p<0.0001 by Mann-Whitney).

(b-e) Unlike C18:0, C16:0 does not induce mitochondrial fusion *in vivo*. A banana milk shake was ingested, containing an equal molar amount of C16:0 corresponding to the 24g of C18:0 used the C18:0 milk shake. Blood was drawn at the indicated timepoints after C16:0 ingestion and (b) the mitochondrial morphology of blood leukocytes was scored as shown in main Figure 1b. C16:0 ingestion does not cause significant mitochondrial fusion compared to the 0h timepoint. n=60 cells per timepoint, 1 subject. (c) Serum C16:0-TAG levels do not increase upon C16:0 ingestion. (d-e) Serum acylcarnitine levels do not drop upon C16:0 ingestion.

(f-h) Unlike C18:0, C16:0 does not induce mitochondrial fusion in vivo. Six healthy subjects from the main study were re-recruited to follow a low-C18:0 diet for two days and were then given a banana milk shake containing either C18:0, or C16:0, or neither, and neutrophil mitochondrial morphology was scored in a double-blind fashion. (f) Mitochondrial morphology does not change in 5 subjects who received a banana milk shake containing an equimolar amount of C16:0 corresponding to the 24g of C18:0 used in the C18:0 milkshake. (60 neutrophils were scored per subject, n=5 subjects, n.s. p>0.05 by student's t-test). (g) Neutrophil mitochondria fuse in one 'positive control' subject in response to C18:0 ingestion (**** p<0.0001 by Mann-Whitney test). (h) Mitochondria do not fuse in one 'negative control' subject receiving a mock drink without added C16:0 or C18:0 (n.s. p>0.05 by Mann-Whitney test). In all cases, blood samples were drawn at 08:45, just before the subjects received respective drinks (0h sample) and at 11:45, 3 hours after ingestion of the drinks (3h sample) and mitochondrial morphology of neutrophils was scored as described in main Figure 1b. The experiment was carried out in a double-blind fashion – the identity of the samples was revealed only after all scoring and analyses were performed. Moreover, a second person repeated and confirmed the scoring, also in a double-blind fashion.

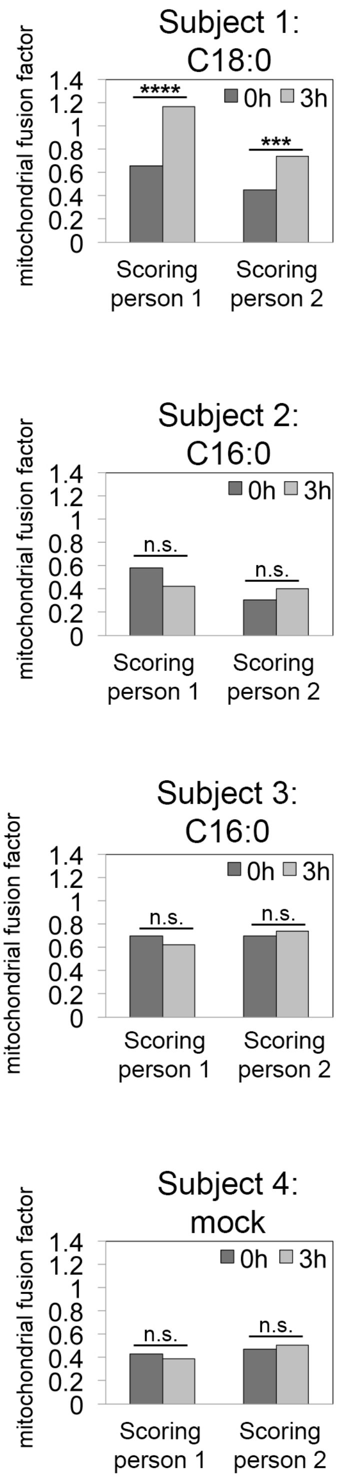

Supplementary Figure 7: Mitochondrial morphology for 4 subjects scored independently by two people.
Results are qualitatively similar for two people independently scoring neutrophil mitochondrial morphology on the same blood samples. The blood samples were prepared as described for Supplementary Figure 6f-h (n=60 neutrophils, **** $p<0.0001$, *** $p<0.001$ of Mann-Whitney test).
