# Supplementary material for: Dietary stearic acid regulates mitochondria in vivo in humans
Source: Nat Commun. 2018 Aug 7;9:3129. doi: 10.1038/s41467-018-05614-6 (PMC6081440; doi:10.1038/s41467-018-05614-6)
Supplement: Supplementary file 3 — Description of Additional Supplementary Files [file 41467_2018_5614_MOESM3_ESM.docx]

**Description of Additional Supplementary Files**

**File Name: Supplementary Data 1**

**Description:**

Baseline Characteristics for all subjects in the study.

**File Name: Supplementary Data 2**

**Description:**

Basic clinical parameters for each subject in the study.

**File Name: Supplementary Data 3**

**Description:**

Multivariate regression analysis (performed with SPSS statistical analysis software) identifies only C18:0-TAG levels (normalized to C16:0-TAG) and no other clinical parameter as significantly affecting the dependent variable, mitochondrial fusion factor.

**File Name: Supplementary Data 4**

**Description:**

Correlations between various measured parameters
